# Nongenomic roles of thyroid hormones and their derivatives in adult brain: are these compounds putative neurotransmitters?

**DOI:** 10.3389/fendo.2023.1210540

**Published:** 2023-08-28

**Authors:** Joseph V. Martin, Pradip K. Sarkar

**Affiliations:** ^1^ Biology Department, Center for Computational and Integrative Biology, Rutgers University, Camden, NJ, United States; ^2^ Department of Basic Sciences, Parker University, Dallas, TX, United States

**Keywords:** thyroxine, iodothyronine, thyronamine, non-canonical, nongenomic, neurotransmitter, thyroacetic acid

## Abstract

We review the evidence regarding the nongenomic (or non-canonical) actions of thyroid hormones (thyronines) and their derivatives (including thyronamines and thyroacetic acids) in the adult brain. The paper seeks to evaluate these compounds for consideration as candidate neurotransmitters. Neurotransmitters are defined by their (a) presence in the neural tissue, (b) release from neural tissue or cell, (c) binding to high-affinity and saturable recognition sites, (d) triggering of a specific effector mechanism and (e) inactivation mechanism. Thyronines and thyronamines are concentrated in brain tissue and show distinctive patterns of distribution within the brain. Nerve terminals accumulate a large amount of thyroid hormones in mature brain, suggesting a synaptic function. However, surprisingly little is known about the potential release of thyroid hormones at synapses. There are specific binding sites for thyroid hormones in nerve-terminal fractions (synaptosomes). A notable cell-membrane binding site for thyroid hormones is integrin αvβ3. Furthermore, thyronines bind specifically to other defined neurotransmitter receptors, including GABAergic, catecholaminergic, glutamatergic, serotonergic and cholinergic systems. Here, the thyronines tend to bind to sites other than the primary sites and have allosteric effects. Thyronamines also bind to specific membrane receptors, including the trace amine associated receptors (TAARs), especially TAAR1. The thyronines and thyronamines activate specific effector mechanisms that are short in latency and often occur in subcellular fractions lacking nuclei, suggesting nongenomic actions. Some of the effector mechanisms for thyronines include effects on protein phosphorylation, Na^+^/K^+^ ATPase, and behavioral measures such as sleep regulation and measures of memory retention. Thyronamines promptly regulate body temperature. Lastly, there are numerous inactivation mechanisms for the hormones, including decarboxylation, deiodination, oxidative deamination, glucuronidation, sulfation and acetylation. Therefore, at the current state of the research field, thyroid hormones and their derivatives satisfy most, but not all, of the criteria for definition as neurotransmitters.

## 1 Introduction

The brain is a key target for the thyroid hormones (THs), since the major symptoms of patients with dysthyroidism are all related to brain-specific functions, such as sleepiness, depression and nervousness (1–4). However, the functions of THs and their derivatives in the adult central nervous system (CNS) are as of yet poorly understood.

As early as the 1970s, Dr. Mary B. Dratman emphasized that thyroid hormones are aromatic amino acid analogs of tyrosine (5, 6). All other known aromatic amino acids are decarboxylated to form biogenic amine neurotransmitters, such as dopamine, norepinephrine and serotonin. She proposed that thyroid hormones could also be decarboxylated and might be therefore be the precursors of catecholamine-like amines (5). Of particular interest in this regard was the observation that the effects of hyperthyroidism resemble a hyperactivation of the adrenergic system and that the β-adrenergic blocker propranolol ameliorates many of the signs and symptoms of thyrotoxicosis (7).

**Figure 1:**
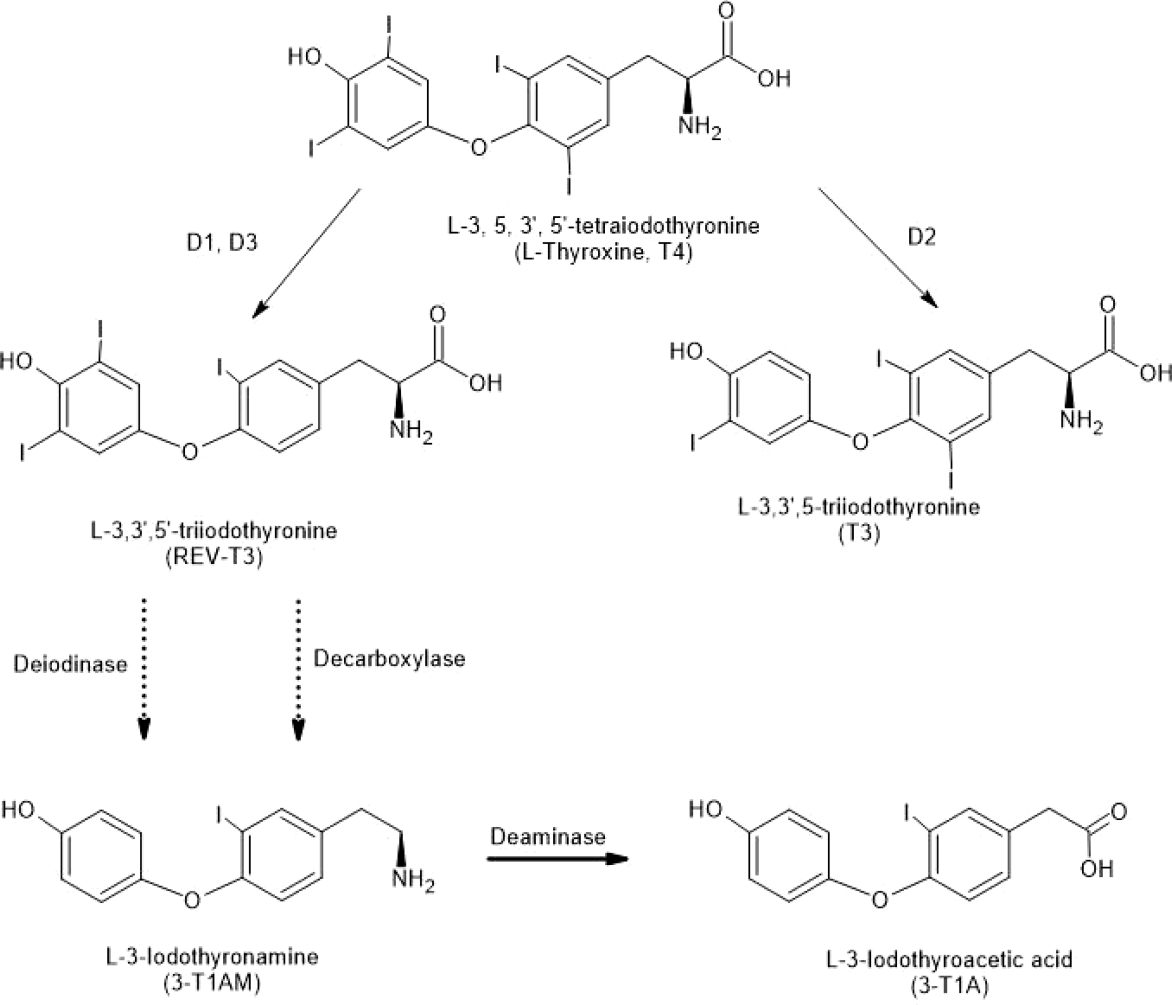
Structures of Thyronines and Derivatives. Deiodinases (D1, D2 and D3) remove iodine moieties from the thyronine structures (top three structures). Decarboxylation of a thyronine results in a thyronamine (see 3-T1AM at bottom of figure). Deamination of 3-T1AM results in 3-T1A. Solid arrows indicate single reactions; the dotted arrows indicate reactions that are not explicitly defined.

Although predicted in 1974, it was not until 2004 that the first decarboxylated thyroid hormone derivative, 3-T1AM, was discovered in brain (8), initiating a sea-change in brain thyroid hormone research (**Figure 1**). Scanlan (8) showed that systemic injections of 3-T1AM lowered body temperature and decreased heart rate. We showed that microinjections of 3-T1AM into the preoptic region of brain also decreased body temperature and had effects on sleep (9). Since injections to the lateral ventricle of brain were ineffective, the effects were localized to brain neuropil itself. Also, 3-T1AM was shown to bind to trace amine-associated receptors (TAARs), particularly TAAR1 (10). However, 3-T1AM’s thermoregulatory effects persist in TAAR1 knockout mice (11). Moreover, abrogation of the binding of the thyroid hormone receptor to DNA still allows thermoregulatory and cardiac effects of the hormone (12). Dratman and coworkers found that microinjections of 3-T1AM into the locus coeruleus changed cell firing rates there (13). Dratman (13, 14) and others (15, 16) suggested the idea that THs or their derivatives can act as neurotransmitters in adult brain.

Neurotransmitters are commonly small, hydrophilic molecules which act extracellularly on membrane-spanning receptor proteins that do not interact directly with the genetic apparatus (17). Such “nongenomic” actions are typical of neurotransmitters. On the other hand, THs have a variety of types of actions (18). Type 1 effects are genomic actions of THs mediated subsequent to binding to canonical thyroid hormone receptors (THRs) which interact with DNA in the nucleus to regulate gene expression. Type 2 effects are also genomic, yet the actions of the THRs are mediated by a secondary protein that, in turn, regulates gene expression. Type 3 effects are mediated by THRs, but there is not an interaction of THR with the chromatin. Type 4 effects of THs are those which do not require the canonical THR or any direct interactions with the genome. Types 3 and 4 TH effects are the nongenomic effects most like neurotransmitter actions. In distinguishing between nongenomic (3 and 4) and genomic (1 and 2) actions, time-course is critical; genomic effects generally require1-2 days to manifest, while nongenomic effects are apparent much more rapidly, on the order of seconds to hours [see (19)]. While some genomic effects of THs do indeed occur in mature brain (20), the current paper will focus on nongenomic effects typical of neurotransmitters.

Neurotransmitters are defined by their (a) presence in the neural tissue, (b) release from neural tissue or cell, (c) binding to high-affinity and saturable recognition sites, (d) triggering of a specific effector mechanism when added to the sensitive brain site and (e) inactivation mechanism (17). The remainder of the current paper will evaluate each of the characteristics of a neurotransmitter with regard to thyroid hormones and their derivatives.

## 2 Presence of TH and metabolites in brain

The thyroid gland, a unique example of a halogenating system in mammals, is the ultimate source of all iodothyronine hormones and their metabolites. THs, secreted from the gland mainly as L-thyroxine (T4), are deiodinated by specific enzymes (deiodinases D1, D2, and D3). Of these, D2 and D3 are localized in brain (21–23). Decarboxylated metabolites of THs, known as thyronamines (TAMs; **Figure 1**), are also concentrated in brain tissue over serum (8, 24). An *in vitro* model of the blood-brain barrier indicated that 3-T1AM is efficiently transported across the barrier, while 3-TA is not (25).

### 2.1 Levels of THs and TAMs in adult rat brain

**Figure 2:**
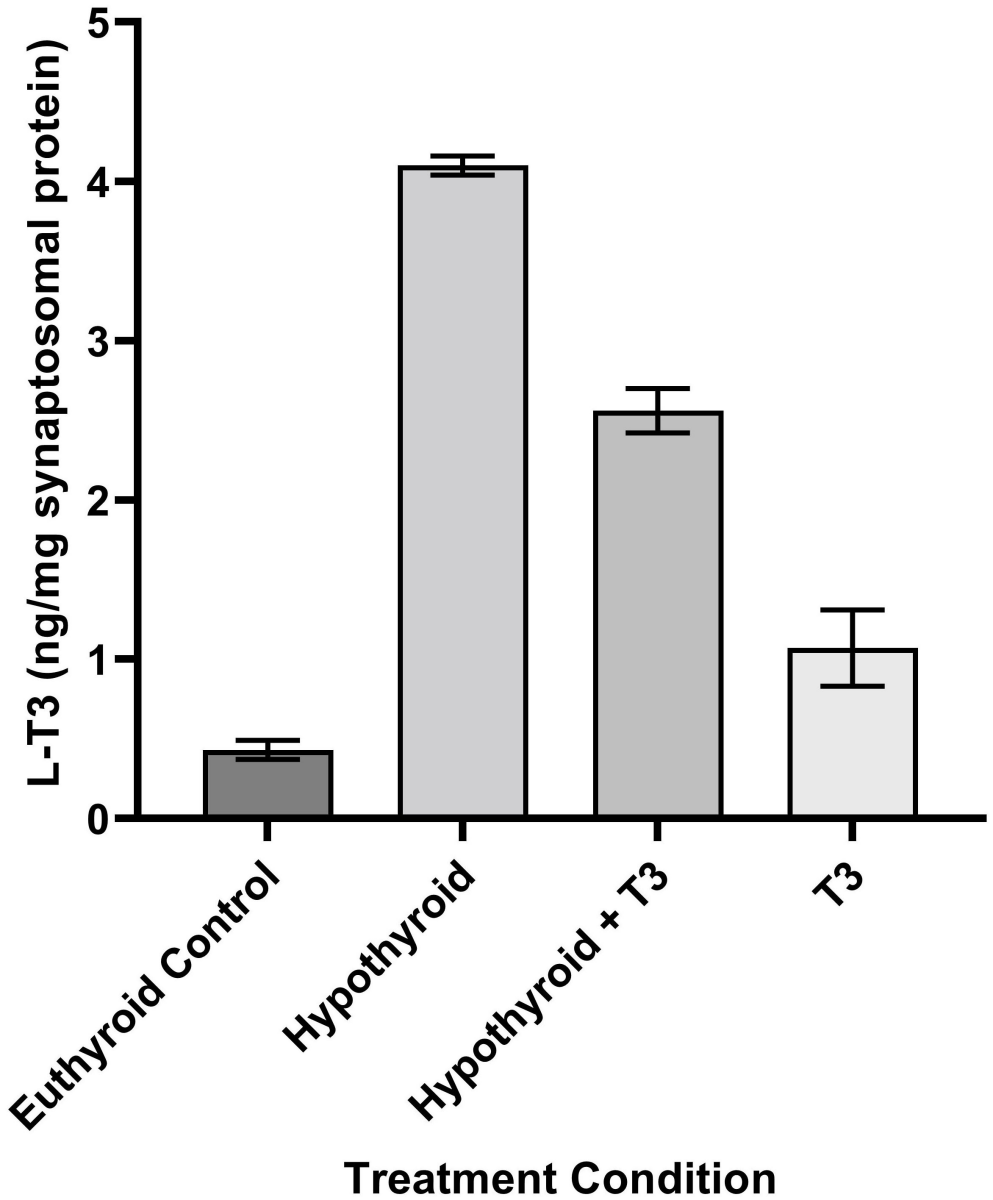
Schematic of the Process of Neurotransmission. Numbers in parentheses refer to citation numbers of papers for THs or their derivatives.

THs and their derivatives selectively accumulate in brain regions over blood (13, 21, 26–29). Tissue levels of TH vary significantly by brain area (30). Levels of TH and derivatives in adult rat brain are in the range of ng/mg protein (**Figure 2**). In mature rat brain cell nuclei, the levels of radiolabeled TH showed a steady decline (14, 21, 27, 29, 31). Hypothyroidism is accompanied by a decrease in 3-T1AM as measured by LCMS in blood and liver extracts (32).

Tissue levels of TH vary significantly by brain area, with L-T3 ranging from 792 fmol/g in amygdala to 2821 fmol/g in midbrain (30), while L-T4 ranges from 1051 fmol/g in cortex to 6255 fmol/g in olfactory bulb (30).

Accumulation of ^125^I-T3 and ^125^I-T4 in adult rat brain was seen following intravenous injection of the compounds (21, 29, 31). Injection of ^125^I-T4 intravenously prior to thaw-mount autoradiography resulted in a saturable localization of radioactivity in distinct brain areas (27). Interestingly this labeled T4 was converted to T3 within the nerve endings by deiodinase activity (**Figure 1**). The hypothalamus and brainstem were elevated in TH in these studies (30). The TH is carried by anterograde axonal transport from the locus coeruleus to terminal projection sites throughout the forebrain (33). Cultured neurons in a microfluidic chamber showed both anterograde and orthograde axonal transport of labeled T3 (34).

#### 2.1.1 Transport of THs and TAMs in adult rat brain

The transport of TH into brain cells is thought to occur in two phases [see (35)]. T4 is transported down its concentration gradient into astrocytes, where it is rapidly deiodinated by D2 to T3. Subsequently, T3 is transported up its concentration gradient into neurons, where D2 is absent (36).

Recently, it has been recognized that there are around 15 transporter molecules that carry THs across cell membranes and into brain cells in a saturable fashion, often depending upon Na^+^ gradients (37). Monocarboxylate transporter (MCT)8 and MCT10, encoded by the *SLC16A2* gene, are some of the most effective transporters across the cell membrane. Organic anion transporting polypeptide (OATP)1C1 is also a highly effective transporter and is encoded the *SLCO1C1* gene. Long-chain fatty acid transport protein 4 (FATP4) is encoded by the *SLC27A4* gene. L-type/large neutral amino acid transporters (LAT) 1-4 are coded by *SLC7A5*, *SLC7A8, SLC43A1*, and *SLC43A2*, respectively. Once transported, T4 is enzymatically converted to its known active form T3 within the CNS by the neuronal D2 activity (38–40).

μ-Crystallin has been recognized as another hydrophobic cytosolic NADPH-dependent TH-binding protein-cum-enzyme in adult brain. Specific T3-binding to μ-crystallin inhibited its activity, whereas the other TH-analogs were without effect. The binding of T3-μ-crystallin has been compared to T3 binding to its nuclear receptor in glial cells (41).

#### 2.1.2 Uptake of TAMs in adult rat brain

TAMs are also selectively increased in brain over blood (26). A brain regional specificity of TAM uptake is suggested by mass spectrometry coupled to liquid chromatography (LCMS) measurements of cortical 3-T1AM nearly three-fold of those in cerebellum (24). After intravenous injection of ^125^I-3-T1AM in rats, the distribution of radioactivity in autoradiographs showed hotspots in various brain areas, including the cingulate, motor and retrosplenial cortices (13). In addition, high levels of radioactivity were seen in the paraventricular and supraoptic nuclei of the hypothalamus and the lateral and medial mammillary nuclei. The cerebellar granule cell layer and the pontine nuclei are also intensely labeled. The numerous hotspots for binding suggest that various brain systems are involved in actions of TAMs. The intensity of the counts against a light background indicates a high neuroanatomical specificity of the binding of TAMs.

### 2.2 Levels of T4 and T3 in subcellular fractions from adult rat brain

**Figure 3:**
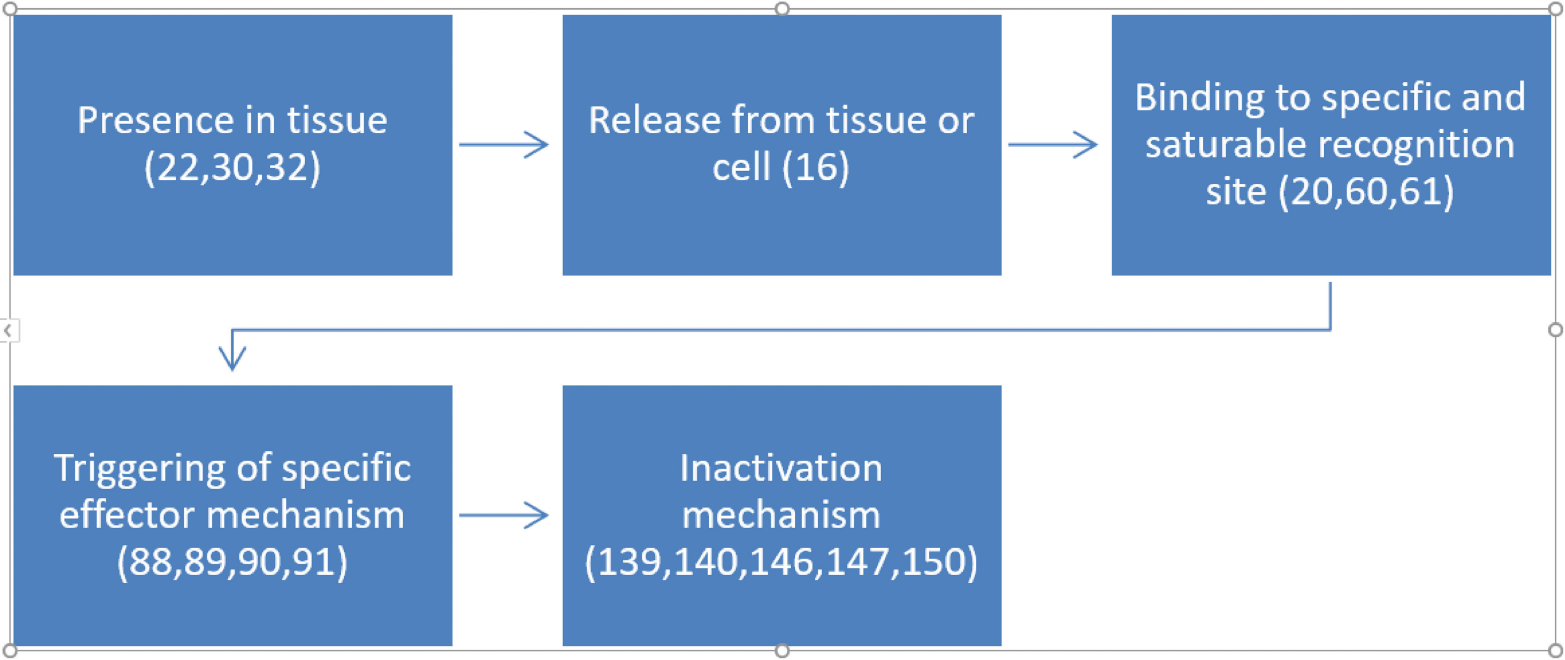
Levels of L-T3 in Synaptosomes from Rat Cerebral Cortex in Various Thyroid States. Hypothyroid: PTU-treated hypothyroid rats brain; Hypothyroid + T3: PTU-treated rat with IP T3 (2 μg/g body weight), T3 (2 μg/g BW). Error bars indicate standard errors of the mean (SEMs). Reproduced, with permission, from (1).

Sarkar and Ray (42) investigated synaptosomal levels of T3 under different thyroidal conditions (42). In N-propylthiouracil (PTU)-induced hypothyroid adult rat cerebrocortical synaptosomal fractions, surprisingly, we noticed higher levels of T3 (~9.5-fold; ~126 nM) in contrast to the euthyroid control values (**Figure 3**). The levels of synaptosomal T4 levels remained undetected. Intraperitoneal (IP) injection of T3 in euthyroid and PTU-injected adult rats showed about ~2.5-fold and ~6-fold higher levels of synaptosomal T3 respectively, compared to euthyroid controls. However, as expected, due to IP injection of additional T3, we detected ~2.5-fold higher concentrations of synaptosomal T3 in the euthyroid + T3 group. Notably, the assay used may not have distinguished between T3 and 3-T1AM.

Detection of low levels of T3 in whole rat brain homogenate could be attributed to this mixed and heterogeneous brain fraction population consisting of several brain areas and other subcellular fractions. Nonspecific binding properties of other intracellular proteins in whole brain homogenate also could be ruled out for the detection of low levels of brain TH (8). Three other supporting investigations also estimated T3 content within the adult rat brain synaptosomal fraction, with similar results as above (15, 42, 43).

Investigators elsewhere also determined similar levels of T3 in different brain areas and at various thyroid status (44, 45). The higher concentrations of neuronal T3 in hypothyroid status can be attributed to the immediate boosted activity of neuronal D2 to counteract the adverse situation produced due to emergency hypothyroid signals in brain. High brain T3 levels might address the potential physiological need for THs during acute brain hypothyroidism (15, 42).

^125^I-T3 is selectively taken up and concentrated in the synaptosomal fraction (21). In addition, hypothyroid animals used by Sarkar and Ray (42) after 14 days of PTU treatment, do not reach the longer term equilibrium observed in the chronic hypothyroid condition used by other workers (15, 44). An inhibitory control mechanism by neuronal D2 activity can be suggested for decreased levels of synaptosomal T3 in the cerebral cortex followed by exogenous IP T3-treatment to the hypothyroid animals. In fact, a decrease in D2 activity was observed within 4 hours of IP T3-injection to thyroidectomized adult rats. This observation, along with a similar report (42) that also found decreased levels of synaptosomal T3 after 24 hours of T3 treatment, suggests an existence of a regulatory mechanisms during various thyroid status. However, the exact mechanism of action remains unclear (21, 45).

T3 levels were also detected by a radioimmunoassay technique within the brain cerebrocortical non-synaptic mitochondria of adult rat brain. While levels of T4 remained undetected within brain non-synaptic mitochondria, T3 levels were demonstrable. In contrast to the synaptosomal fraction, the T3 levels within the nonsynaptic mitochondria, prepared from adult rat brain cerebral cortex was found to be ~3.2-fold more (46).

After T4 enters the brain (predominantly in glial cells), its outer ring can be deiodinated by D2, forming T3 or the inner ring can be deiodinated by D3, forming 3,3’,5’-triiodothyronine (rT3) (47, 48) (**Figure 1**). T3 is highly active in genomic actions in most tissues, while rT3 is largely inactive (49). According to Scanlan’s group (36), starting with rT3, two successive outer ring deiodinations by D2, with a decarboxylation step, would result in the thyronamine 3-T1AM (**Figure 1**). TAMs can be oxidatively deaminated to various thyroacetic acids (TAs) (50–52).

Although the transportation and accumulation of radiolabeled T4 and T3 were noticed along with its enzyme-catalyzed conversion to neuronal T3 within the neuronal endings, several experiments conducted by various investigators were unable to detect a significant amount of brain T4. A rapid conversion of T4 to T3 by neuronal D2 would explain the lack of TH, although this was not confirmed yet. However, both the nonsaturable and saturable uptakes of T4 and T3 were observed respectively in synaptosomes in *in vitro* models. This clearly indicated existence of two different uptake systems for THs (21, 45, 53, 54).

## 3 Release of TH and derivatives from neural tissue

Although Ca^2+^-dependent depolarization-induced TH release from synaptosomes has been reported (15), this result has not yet been reproduced. A problem may be a lack of sensitivity in assays for the compounds (**Figure 3**).

Studies of TAM release from neural tissue are also rare. The adenosine triphosphate binding cassette (ABC) transport protein, P-glycoprotein (P-gp, or ABCB1) is a potential release mechanism for TAMs (55).

3-T1A is postulated to have exporters involved in release from cells, yet little is known about the nature of the proteins (56).

## 4 TH and metabolite binding in brain

T3 specifically binds *in vitro* to at least two distinct sites in synaptosomes (22, 57–59), which might be responsible for modulatory effects on known neurotransmitter receptors or transporters, or correspond to a novel specific membrane receptor for TH in brain (**Figure 3**).

### 4.1 TH recognition sites in neuronal membrane

The presence of functional nuclear receptors in brain is well-known in developing animals. High affinity and low-capacity nuclear receptor isoforms for TH also have been described in adult brain (60, 61). However, no functional properties could be assigned properly for these brain nuclear isoforms in adult mammalian brain. Scatchard plot analysis also revealed two classes of specific T3-binding sites in the synaptosomes prepared from adult rat brain (19, 59) and chick embryo (62). One of the T3 binding sites exhibited high−affinity, low−capacity and the other with low affinity, high−capacity. Recently, membrane receptors for THs have been described in the majority of cells (62). These receptors have been identified as integrin αvβ3. (Please see articles by Davis elsewhere in this issue.)

T3AM was used to examine its potency to inhibit specific ^125^I-T3 binding in synaptosomes with the idea that it may be a decarboxylated product of T3 and may have actions like T3. The ED_50_ value for T3AM was determined as 10 nM (59). Although T3AM levels in synaptosomes are not known as of yet, this ED_50_ is in the range of concentrations of active THs in brain. It would be of value to measure T3AM in synaptosomes to consider whether this compound might have a physiological role in modulating TH effects in the CNS.

### 4.2 Binding of TH and neurosteroids at GABA_A_ receptors

THs have influences on defined membrane receptors which resemble actions of some neuroactive steroids occurring in brain, called neurosteroids (63). “GABA-positive” compounds increase the activity of the GABA_A_ receptor at nM concentrations. These steroids include 3α-hydroxy-5α-pregnan-20-one or allopregnanalone (ALLOP) and 3α,21-dihydroxy-5α-pregnan-20-one (THDOC) (60, 61). “GABA-negative” neurosteroids, such as pregnenolone sulfate (PREGS), decrease the activity of GABA_A_ receptors at µM concentrations of hormone in much the same way as THs do (64–66).

Although some effects of THs are seen at nM concentrations and nM concentrations of TH were measured in synaptosomes, the concentrations of TH within the synapse following various means of administration of the TH have not been conclusively determined as of yet. Fresh determinations using newer techniques may yield more accurate measurements. Furthermore, the effects of higher concentrations of TH may have relevance for the actions of TH in dysthyroidism, when much higher concentrations of the THs could occur. Finally, so-called pharmacological effects of THs might be important for clinical applications, such as the antidepressant activity of high doses of TH (67–71).

THs inhibit brain binding of ^3^H-GABA (72).

Micromolar concentrations of T3 inhibit GABA-stimulated responses in α2β1γ2 GABA_A_ receptors expressed in *Xenopus* oocytes (73). The GABA_A_ receptor is a pentameric ligand-gated ion channel (pLGIC) or Cys-loop receptor and regulates Cl^-^ currents. Ivermectin (IVM) is an enhancer of GABA_A_ responses (74), and is used as a ligand of the GABA_A_ receptor for crystallization studies, defining the precise localization within the receptor (75). The IVM binding occurs in the transmembrane intersubunit interface of homopentameric *C. elegans* glutamate-gated chloride channel alpha (GluCl) which is used as a prototypical example of pLGICs such as GABA_A_ receptors. The IVM caused increases in chloride currents which were also inhibited by TH in Xenopus oocytes in a competitive way (73). Similarly, ALLOP inhibited the currents in a competitive fashion.

These receptors are also modulated by a variety of agents, including hypnotic drugs (such as benzodiazepines and barbiturates), operating at a variety of distinctive recognition sites within the receptor [see (61, 76)]. We found that low micromolar doses of THs inhibited several activities related to the binding of the GABA_A_ receptor (16, 77).

### 4.3 Effects of TAMs on TAARs

TAARs bind “trace amines” like tyramine, and also amphetamines (78, 79). TAMs bind to the primary binding site of TAAR1 receptors and act as inverse agonists (80, 81). The localization of TAAR1 is in the amygdala, hypothalamus, ventral tegmental area, hippocampus, dorsal raphe nucleus, the nucleus of the stria terminalis, and layer V of the prefrontal cortex (78, 82). TAMs generally have opposite effects on effector mechanisms as compared to THs. TAMs decrease, while THs increase, the body temperature (see section 5.6). However, in TAAR1 knockout mice, the effects of 3-T1AM on thermoregulation persist (11), demonstrating that the TAAR1 receptors are not essential for 3-T1AM control of body temperature. In brain tissue from patients with multiple sclerosis, as compared to controls, TAAR1 protein was seen in microglia and macrophages near the edges of disease-related lesions (83).

TAAR5 receptors are also a target for 3-T1AM and are localized in brain sites, including especially amygdala and olfactory bulb (84). Other sites include some areas of the diencephalon, including the paraventricular nucleus, anterolateral hypothalamus, arcuate nucleus, dorsal lateral geniculate of the thalamus, and zona incerta. TAARs 2, 5, 6, 8, and 9 are all considered to play olfactory functions and act as odorant receptors (78, 85). The receptors are expressed throughout the olfactory system. Although the physiological significance of a role of 3-T1AM in odor sensation is of considerable interest, the data do not support TAAR involvement in the thermoregulatory effects of 3-T1AM.

3-T1AM was associated with G-protein coupled receptors and transient receptor potential (TRP) channels in various cell populations. 3-T1AM has been shown to activate cFOS in the cell cultures prepared from mouse hypothalamic paraventricular nucleus within 60 minutes of *in vivo* administration. However, only a small significant effect was observed on stimulatory G-protein (G_s_)-adenylate cyclase system, whereas no effect was noticed on inhibitory G_αi/o_-adenylate cyclase system. The effect of pharmacological levels of 3-T1AM (10 μM) on cFOS activation was attributed to intracellular Ca^2+^ levels and whole-cell current (86).

### 4.4 Effects of THs on binding to other neurotransmitter receptors

We found that nicotinic acetylcholine (ACh) receptors (nAChRs) isolated from Torpedo electric organ and stimulated with ACh were promptly inhibited by micromolar concentrations of T3 (87). In an analogous way, ACh-stimulated currents in SH-SY5Y neuroblastoma cells are immediately inhibited by T3 or T4 (88). In the nAChRs from Torpedo, the T3 and PS showed a similarity in actions that correlated with a structural similarity between the two compounds (87). The effects of pH were also comparable with respect to the PS or T3 inhibitions of ACh-stimulated currents in the nAChRs. In all, there are striking similarities between the effects of T3 and PS at the nAChR, potentially indicating a commonality in mechanism and site of action. In each of 5 types of muscarinic receptor stably transfected in Chinese Hamster Ovary (CHO) cells, N-methylscopolamine (NMS) binding at muscarinic sites was inhibited in a dose-dependent manner by T1AM (89).

Fifty µM THs inhibit binding of ^3^H-glutamate to brain membranes, especially binding blocked by N-methyl-D-aspartate (NMDA) (72). T3 or T4 quickly inhibit NMDA-evoked currents in rat hippocampal cultures with potency in the micromolar range (90). Inhibition of protein kinase C (PKC) did not alter the inhibitory effect on NMDA-stimulated currents, indicating that the TH effect was not secondary to phosphorylation of the receptor.

Although not in CNS, but with potential relevance, THs in isolated fat cells were found to increase binding of ^3^H-norepinephrine or ^3^H-isoproterenol, the β-adrenergic agonist (91). T3 was more effective than T4. Since this observation was due to an influence on K_d_, and not B_max_, the effect was likely an allosteric modulatory one. In rats made hypothyroid by treatment with PTU in drinking water, α1 receptor binding increased and β2 binding decreased in hippocampus (92). However, since these treatments were over a long period of time, the results might have been due to genomic mechanisms to regulate synthesis of receptor proteins.

Also not in brain, 3-T1A uptake to H9c2 cells (from cardiomyocytes) was saturable and of high-affinity and blocked by iproniazid, the monoamine oxidase (MAO) inhibitor (24). MAO is a likely enzyme for conversion of TAMs to thyroacetic acids (TAs), thereby further supporting the idea that the presence of 3-T1A is required for some effects.

## 5 Initiation of a specific effector mechanism

THs promptly decrease Na^+^-K^+^-ATPase (NKA) activity (93), inhibit GABA-stimulated Cl^-^ flux (16, 66), inhibit nicotinic receptor-related currents (87, 88), increase depolarization-dependent Ca^2+^ uptake (94), and enhance phosphorylation of protein in a nucleus-free brain preparation (95). Mitogen-activated protein kinase (MAPK) is activated by THs (96). Both 3-T1AM and T3 have complex acute effects on sleep and thermoregulation (9, 97, 98). The immediacy of the effects suggests nongenomic mechanisms. In addition, many of the *in vitro* studies were performed in the absence of nuclei, thereby confirming a nongenomic, non-canonical effect.

### 5.1 Nongenomic effects of THs on protein phosphorylation

In non-neural tissues, TH has been shown to nongenomically regulate MAPK (96, 99, 100). We have found that *in vitro* incubation of lysates of rat brain synaptosomes with nanomolar concentrations of T3 alters phosphorylation of several proteins within minutes (95, 101). Both T3 and T4 produced these effects, but not rT3. One of the four most heavily phosphorylated proteins in the synaptosomal lysate had a molecular weight of 113 kDa, approximating the molecular weight of the α subunit of the NKA. (See Section 5.2.) The dose-response curve for the effect of THs was an inverted U-shape, with the highest and lowest concentrations having minimal effects (95). THs activate extracellular signal-regulated kinase (ERK) (102). T1AM induced protein phosphorylation in neuroblastoma cell lines (103), and this effect was not due to metabolic conversion to TAs, which had distinct effects. These data support the contention that THs and derivatives activate a variety of rapid metabotropic pathways for regulation of protein phosphorylation in adult brain of mammals.

### 5.2 Functional correlation of neuronal Na^+^-K^+^-ATPase specific activity and specific T3-binding in the synaptosomal membrane of adult rat brain

NKA is an important membrane spanning enzyme required primarily for maintenance of ion gradients (including those for Na^+^ and K^+^ ions) across the membrane. THs have profound regulatory influences on this major enzyme. The Na^+^ and K^+^ gradients set up by NKA activity are important for establishment of resting membrane potentials, action potentials and transport of certain molecules. Studies have shown that release of acetylcholine (104) and norepinephrine (105) from rat brain cerebrocortical neurons can be regulated by modifying NKA activity. A depolarizing effect that decreases the K^+^ gradient causes neuronal release and subsequent presynaptic re-uptake for these two neurotransmitters (104).

*In vitro* T3 binding correlates with an inhibition of NKA activity in synaptosomes of adult rat cerebral cortex. Addition of T3 (10^-12^ to 10^-7^ M) within 10 minutes of incubation caused a dose-dependent inhibitory response to NKA activity. Such immediate action of T3 *in vitro* was suggestive of a rapid nongenomic action of T3 on the synaptosomal membrane NKA. Further inhibition of NKA activity correlated with increasing binding of ^125^I-T3 to specific T3-binding sites in synaptosomes.

To examine the specific binding of T3 to the synaptosomal membrane, we also used T3-amine (T3AM) in addition to other TH-analogues. The relative order of potencies of binding affinities for the synaptosomal T3 binding sites in the presence of different T3-analogues were as follows: T3>T3AM>T4=TRIAC>r-T3>3,5-T2. For comparison, the rank order of the potencies of the compounds to inhibit NKA activity was T3>T3AM>T4>TRIAC>r-T3>3,5-T2. The concentrations of TH analogues required to displace 50% specific binding (ED_50_ value) of ^125^I-T3 to its synaptosomal binding sites were 10-, 63-, 63-, 1000- and 6250 nM, respectively. The present investigation demonstrated a dose-response correlation between the inhibition of synaptosomal NKA activity and corresponding T3-binding to the synaptic membrane fraction in adult rat brain cerebral cortex (59).

*In vitro* application of brain physiologic concentrations of T3 revealed a correlation between dose-dependent inhibition of NKA activity and increased T3-binding at the high affinity site. In the study, use of 0.1 nM T3 showed ~35% inhibition of the NKA activity that corresponded to ~74% T3-binding. This trend of NKA inhibition and T3-binding was noticed with the following concentration range of T3: 0.5 nanomolar - 10 micromolar. This finally corresponded to ~80% (maximally) T3-binding. This ~80% saturation binding moved towards the low-affinity binding site from the high-affinity binding sites. *In vitro* addition of higher concentrations of T3 (15 micromolar used to determine nonspecific binding) was not able to saturate this low-affinity binding. This inhibition of the NKA activity was nicely correlated up to 0.5 nM T3 and refers to high-affinity binding only. The role of numerous other nonspecific neuronal proteins can be implicated for this nonspecific T3-binding (63).

T3AM was employed to broaden the spectrum of compounds tested for activity to alter ^125^I-T3 binding in synaptosomes. T3AM inhibited label bound to synaptosomes with an ED50 of 10 nM. T3AM decreased synaptosomal NKA activity to 51% of that seen after T3 treatment. Similarly, this dose of T3AM was 71% as effective as T3 to increase Ca^2+^-ATPase activity in human RBCs (62). An increase in mRNA levels for NKA of α, α+ and β-subunits was seen in the developing brain (105), and kidney cortex (106) of rat. However, in adulthood, the NKA activity was seen to not have this responsiveness to T3 in subunit, indicating that T3 acts through nongenomic mechanisms in mature brain.

Patch-clamp techniques also demonstrated the presence of rapid nongenomic T3-dependent mechanisms for neuronal excitability in cell cultures from postnatal rats at various thyroid situations. A significant increase in inward Na^+^ current, and outward K^+^-current was seen in cultured hippocampal and cortical neurons when given a single dose of T3 (30 nM). Hyperthyroid conditions showed higher greater effects in contrast to the hypothyroid animals (107).

### 5.3 Polymerization of actin

Dependency for TH during a critical period of brain development mediated through gene expression is well confirmed. However, nongenomic control of actin polymerization and its active interaction with a basement membrane protein, laminin, in the presence of TH within astrocytes are interesting (108).

### 5.4 Effects of THs and neurosteroids on GABA_A_ receptor-modulated currents

In studies using two-electrode voltage clamp of *Xenopus* oocytes expressing α2β1γ2 GABA_A_ receptors, we showed that PREGS and T3 compete for a binding site (73). Molecular dynamics studies indicated an IVM binding site at the intersubunit interface, which simulations identified as the likely shared site of PREGS and T3 binding (73). As with TH, PREGS levels range in tens of nmol/kg wet weight of brain. Concentrations of PREGS vary with the stage of the mouse estrous cycle (109). Since the onset of an inhibitory effect of PREGS on GABAergic single-unit activity is very sluggish, the steady-state block of receptor function was calculated to occur in the endogenous range of PREGS concentrations (110). Since TH affects the GABA_A_ receptor like PREGS does, as suggested by the three-dimensional structural similarities between the hormones (73, 75, 111), physiological endogenous levels of THs (or elevated levels due to hormone imbalance) in the synapse might contribute to a modulation of receptor function through an action at the intersubunit interface

### 5.5 Effects of THs and TAMs on thermoregulation

The initial report of endogenous TAMs also showed rapid effects of intraperitoneal injection to decrease body temperature in euthyroid mice (8). Subsequently, we microinjected µg amounts of 3-T1AM into the rat preoptic region (9). The intermediate doses of 3-T1AM inhibited core body temperature. These results on thermoregulation were consistent with the general notion that TAMs have opposite effects to THs. The effect was unlikely to be due to metabolism of the 3-T1AM to 3-T1A, since IP injections of up to 50 mg/kg 3-T1A failed to alter body temperature (112). Furthermore, Hones et al. (12) produced knockin mice with a mutation in the nuclear-type TH receptor DNA-binding domain that prevents DNA binding, thus leading to complete loss of genomic TH action. However, the knockin mice still demonstrated a decrease in body temperature subsequent to administration of 3-T1AM. These studies strongly support the contention that influences of 3-T1AM on thermoregulation are direct nongenomic effects.

### 5.6 Effects of THs and neurosteroids on cholinergic effector mechanisms

We found that T3 or PREGS can rapidly inhibit cholinergic currents in Xenopus oocytes expressing nicotinic receptors isolated from Torpedo electric organ (87). Both T3 and PREGS have similar consequences with regard to desensitization of nAChRs and these effects are influenced by pH in similar ways, suggesting a common mechanism of action. In patch clamp studies of SH-SY5Y HeLa cells, both T3 (IC50 = 4.6 µM) and T4 (IC50 = 4.8 µM) immediately inhibited nicotinic receptor currents (88). (In this system, the effective doses seem high and non-physiological. Several other systems show sensitivities in the µM range. While we can measure synaptosomal TH, we do not know the concentration in the synapse. In addition, pharmacological effects can be useful clinically.) Interestingly, T3 inhibited kainic acid currents in cultured hippocampal cells, while up to 20 µM concentrations of T3 or rT3 were without effect. This effect was not blocked by TETRAC, an inhibitor of the integrin αvß3 receptor, suggesting a direct action elsewhere, presumably on the nicotinic receptors.

T3 also inhibits the brain binding of the muscarinic cholinergic ligand ^3^H-muscimol (113).

A decrease in cholinergic excitability was noticed in adult-onset hypothyroid mouse, with deficits in learning and memory (112) (See Section 5.7).

We showed that an acute administration of T3 enhances acetylcholinesterase in synaptosomes prepared from the cerebral cortices of the PTU-induced hypothyroid rats (114). The increase in metabolism of acetylcholine was accompanied by enhanced Mg^2+^-ATPase activity, representing an increase in the uptake of acetylcholine to the intrasynaptosomal synaptic vesicles. Thus, inhibition of nAChR by TH is associated an increase in metabolism of acetylcholine.

### 5.7 Effects of TH and derivatives on learning and memory

Hyperthyroidism causes deficits in learning and memory (Y-maze, novel object, and Morris water maze behavioral assays) which are associated with decreases in mature spines in the hippocampus (115). These effects of hyperthyroidism can be reversed by increasing NMDA and AMPA receptor activity. A decrease in cholinergic excitability was also noticed in adult-onset hypothyroid mice with deficits in memory (116). Conversely, hypothyroidism inhibits an associative memory task using the eye blink response (117). Three daily injections of 500 µg/Kg/day of T4 improve the performance of thyroidectomized rats in spatial memory tasks. Different memory tasks will give different outcomes, with an inhibitory avoidance task being less sensitive to THs than a spatial memory test (118). Effects of TH on spatial memory tasks were correlated with effects on NKA activity. The effects of TH on learning and memory have an inverted U-shaped dose-response curve, with both high and low doses causing decrements.

The documented correlations of TH effects on learning and memory to ionotropic receptor activity or NKA activity point to potential nongenomic mechanisms of action. The typical administration of THs is chronic (i.e., 20 days) in these studies, consistent with a predicted genomic effect. To determine nongenomic effects, it will be important to determine the influence of more acute injections on influences of THs on measures of spatial memory.

Intracerebroventricular (ICV) injections of µg quantities of 3-T1AM also have facilitatory effects on learning and memory tasks, including object recognition tests (102). The pain threshold was also increased. Some of these effects may have been due to 3-T1A, the primary oxidative product of 3-T1AM (119).

### 5.8 Effects of thyroid hormone and metabolites on sleep

One of the major symptoms of hyperthyroidism is insomnia (3, 4). The complaint of hypersomnolence is frequent in hypothyroidism (1) and is the presenting symptom in some patients (2). In studies of the effect of severe chronic sleep deprivation, one of the most consistent findings has been a drop in levels of THs (120–123). EEG studies have generally confirmed that during dysthyroidism in humans, there are significant changes in sleep measures. Similar results were noted in animals after experimental manipulations of thyroid activity, yet there has been little agreement as to the details (124–127). Gull et al. (128) found that in rats made hypothyroid by treatment with chronic PTU, there were significant increases in the amplitude of EEG waves, not only in nonREM sleep (NREMS) but also in total sleep and waking (as compared with sham-operated controls). Salin-Pascual et al. (129) demonstrated a significant increase in delta sleep within NREMS in thyroidectomized rats as compared to euthyroid controls.

**Figure 4:**
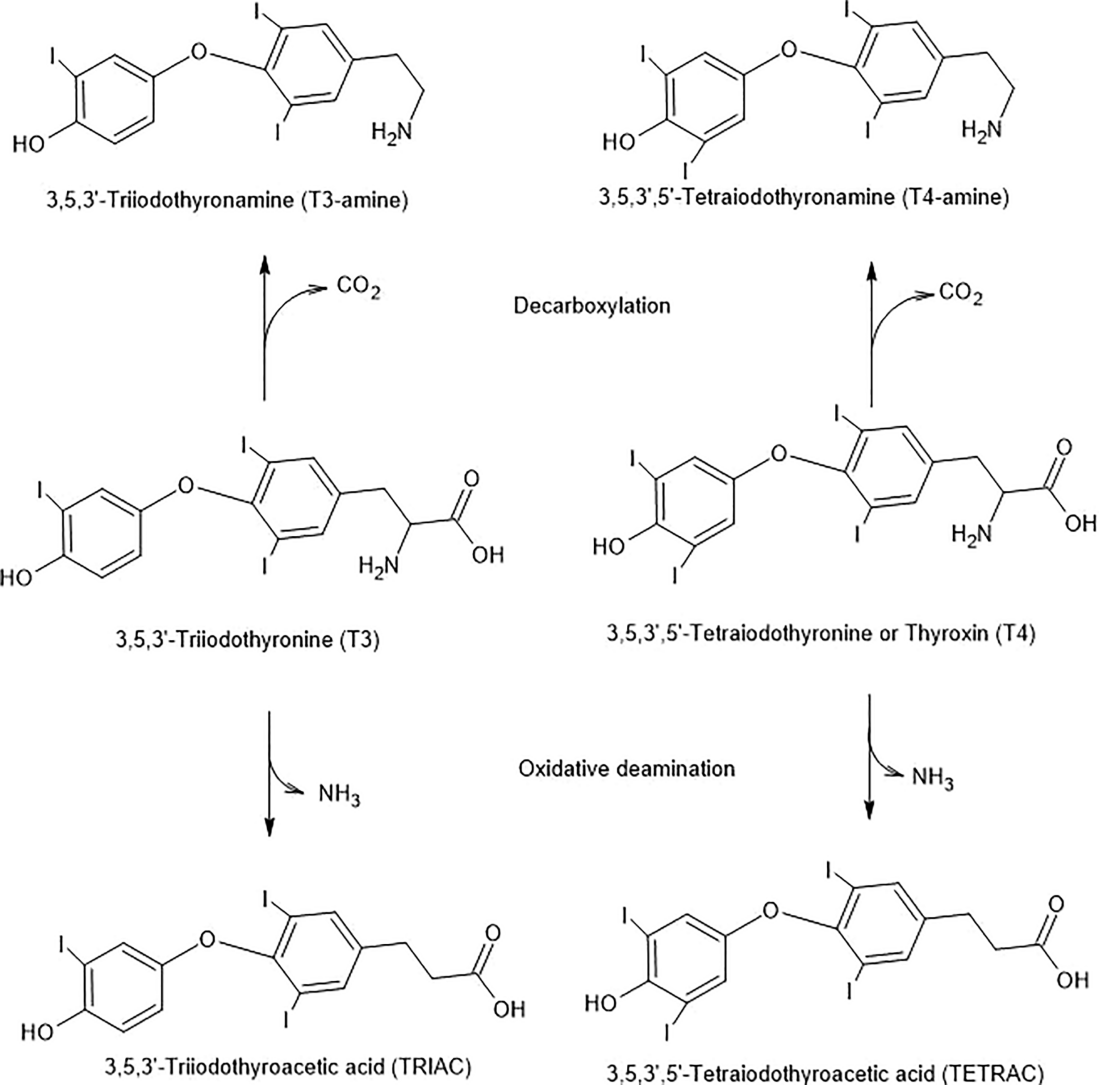
Potential T3 and T4 Inactivation Pathways. The pathways are initiated by decarboxylation or oxidative deamination and are combined with deiodinase steps.

We demonstrated that either hypothyroid (97) or euthyroid (98) rats showed similar patterns of sleep following bilateral microinjection of T3 in the µg range to the medial preoptic region. (The term “medial preoptic region” took into account the likely spread of the microinjection and includes the medial preoptic area, ventrolateral preoptic nucleus, and median preoptic nucleus.) NREMS showed a U-shaped dose response curve. Significant inhibitory effects on NREMS were noted in the intermediate doses (1 or 3 µg T3) in contrast to the lowest (0.3 µg) and highest (10 µg) doses. Waking time was significantly enhanced at the intermediate doses (1 or 3 µg T3) in both hypothyroid and euthyroid rats. However, REM sleep (REMS) was enhanced in euthyroid rats, but inhibited in hypothyroid rats. Injections of TH into the ventricular system were ineffective, suggesting that the hormonal influences were likely due to a direct action on the neuropil, and not to diffusion from the adjacent ventricles. Except for the effects on REMS, the results for hypothyroid or euthyroid rats were quite similar, suggesting some common mechanisms of action. It would be tempting to think that the effects of T3 are due to the decarboxylation of the compound to form 3-T1AM for some aspects of T3 action (**Figure 4**). However, also see section 5.7.

In 1987, we showed that IP injections of THDOC, the GABA-positive neurosteroid, enhanced NREMS (130). Later studies confirmed effects of THDOC on NREMS and demonstrated effects of another GABA-positive neurosteroid, ALLOP, to enhance slow-waves within NREMS (131). Peripheral injections of progesterone also had positive effects on NREMS (132)

The dose effects of TH may exert important effects on the sleep-regulatory system as a neuromodulatory agent resembling a GABA-negative neurosteroid like PREGS (16, 111).

### 5.9 Effects of THs and TAMs on depression

Depression is commonly associated with hypothyroidism (133–136). About 10% of the depressed population has subclinical hypothyroidism and an additional 35% have a reduced TSH response to TRH. Experimentally induced hypothyroidism in rats is associated with increases in turnover of serotonin in the brainstem, suggesting a mechanistic connection between thyroid hormone action and the brain serotonin system (137).

In early studies, high doses of THs were found to relieve depression (67–71). In patients treated with high doses of T4, depression relief persisted despite abatement of anxiety-related symptoms over time (70). T3 enhances the antidepressant effectiveness of T4 treatment (67) and speeds the therapeutic effect of tricyclic antidepressants (138). Most of these experiments involved chronic treatments, leaving open the question of whether the effects could be due to nongenomic actions.

Recently, it has been shown that ALLOP can decrease measures of depression in a rapid acute fashion (139), consistent with a nongenomic action. Given the parallels between binding and actions of TAMs and neurosteroids, it is possible that TAMs might also have nongenomic effects on depression.

### 5.10 Effects of TAs on scratching responses and pain responses

Subcutaneous injection of low µg/kg doses of 3-T1A induced scratching behavior in mice within 10 min, and reduced the sensitivity of pain responses to a heated hotplate within 15 min (140). Western blot analysis of proteins from the dorsal root ganglion indicated an increase of phosphorylated ERK1/2 following 3-T1A. The dose-response curve was an inverted U-shape, with increased activity at 0.4 or 1.32 µg/kg doses and little to no activity seen at 0.1 or 4 µg/kg doses. The effects of 3-T1A were not evident in histidine decarboxylase knockout mice, supporting the hypothesis that these effects are due to an influence on histaminergic neurotransmission. The effect of 3-T1A to induce scratching was abolished by pretreatment with pyrilamine, the inverse agonist at the H_1_ receptor site.

### 5.11 Effects of THR outside of the chromatin (type 3 effects)

A crosstalk between nongenomic action of TH and genomic actions also occurs.

Our major focus in this review paper is on the nongenomic action of TH, in particular in adult mammalian brain. However, a rapid nongenomic connection between the cytosolic TRβ isoform and PI3K signaling was noted following 100 nM levels of THs within 1-5 minutes in pituitary derived GH_4_C_1_ cell culture. Subsequent nuclear translocation of TRβ was found. Similarly, dissociation of TRβ from the p85-TRβ complex was also noticed in CHO cell cultures in the presence of T3 (0.1-100 nM), *in vitro*. T3-induced PI3K activity caused tyrosine phosphorylation of the SH2-domain of the TRβ isoform (141).

Furthermore, the same research group illustrated a rapid DNA-independent significant activation of voltage-gated potassium-channel protein component, KCNH2, in the presence of the nM T3, by the nuclear TRβ2, through PI3K pathway in rat cultured pituitary GH_4_C_1_ cells, a subclone from GH3 pituitary tumor cells. An association of the TRβ2 isoform with the regulatory p85 subunit of PI3K was thus recognized once again for this rapid non-transcriptional action These studies further demonstrated an association of nongenomic cytosolic/membrane events to nuclear-mediated genomic mechanisms of action. Other studies also reported activation of PI3K signaling by TRα. Thus, TH-signaling *via* PI3K was implicated in synaptic maturation and plasticity in mouse post neonatal hippocampus (142).

Other studies using cultured human and bovine endothelial cells also indicated T3-induced association of the TRα_1_ isoform with p85 of the PI3K. This T3-dependent association caused significant phosphorylation of Akt at its serine reside and eNOS phosphorylation using nanomolar concentrations of T3 (1 -100 nM) (143).

Dependency for TH during a critical period of brain development mediated through gene expression is well confirmed. However, nongenomic control of actin polymerization and its active interaction with a basement membrane protein, laminin, in the presence of TH within astrocytes are interesting (108).

## 6 Inactivation mechanisms

TAMs are sensitive to monoamine oxidase (MAO). For example, with T1AM as the substrate, the product is 3-T1A (52) (**Figure 4**). However, as mentioned in section 5.9, 3-T1A has effects on itch behavior and pain responses through influences on histaminergic neurotransmission. Brain-specific deiodinases also inactivate THs (144) and TAMs (36, 145).

The metabolites of THs can be produced mainly by (a) deiodination, (b) deamination, (c) decarboxylation, (d) acetylation, (e) glucuronidation and (f) sulfation in specific tissues by specific enzymes. Products are excreted in the urine and feces.

### 6.1 Deiodination as an inactivation mechanism

Deiodination of iodothyronines is a primary mechanism to control TH action. Deiodinase isozymes catalyze TH synthesis and further metabolism to eventually form inactive metabolites (38, 39).

Three key selenocysteine isoenzymes, commonly called monodeiodinases D1, D2 and D3, are implicated in TH metabolism. D1 is the main deiodination enzyme in the peripheral tissues. The catalytic action of D1 causes inner ring deiodination of T4 to form r-T3, as well the outer ring deiodination to form T3. D2 enzymes have been localized in many brain areas. This not only includes glial cells and astrocytes, but also tanycytes. D2 gene expression was also found to be dependent on neuronal uptake of the blood levels of T4 and its conversion to T3. Thus D2 gene expression in neural tissues implicate possible important protective mechanism to challenge hypothyroid signals in brain, and thereby restore homeostasis.

Conversion of T4 to T3 by both D1 and D2 is suggested for maintenance of tissue homeostasis (39).

Both D1 and D2 can catalyze the conversion of another TH metabolite, rT3 into 3,3’-T2.

The gene expressions for these deiodinases are not only tissue-specific during the developmental stages and adulthood, but also operate in a synchronized pattern, and are cued by central energy expenditure as well as by clinical or disease states, such as in hypoxic situations leading to control of metabolic mechanisms (40).

D3 solely catalyzes the conversion of L-T4 to rT3, and T3 to 3,3’-T2. D3 catalyzed reactions predominantly occur during embryonic period. Still, in adulthood D3 is present in the CNS, and skin (39).

Possible deiodination of TAMs is also described *in vitro*, such as T3AM to 3-T2AM and subsequently to 3-T1AM and finally to T0AM (146). Although several reports of TAM biosynthesis have been published, still limited information is available to specify the exact mechanism(s). The role of monoamine oxidase B and semicarbazide-sensitive amine oxidase was also described for the synthesis of TAM-metabolites. In addition, D1 also causes deiodination of thyroxine sulfates, into physiologically inactive T3S (38).

### 6.2 Deamination as an inactivation mechanism

Deamination of T4 and T3 can produce their respective metabolites, TETRAC and TRIAC. These reactions are catalyzed by amine oxidase (**Figure 4**).

TRIAC has been used clinically as a blocker of TH effects in thyrotoxicosis (147, 148). TETRAC is an inhibitor of the integrin αvß3 receptor (149, 150). Deamination inactivates THs, and, additionally, the new compounds have inhibitory effects on the TH receptor binding and activities.

One of the recently discovered oxidatively deaminated endogenous products of 3-T1AM, is 3-iodothyroacetic acid (3-T1A) (50) which has effects mediated by histaminergic neurotransmission (see Section 5.9). Therefore, the effects of deamination of THs and TAMs are not simple inactivations of the compounds but also inhibitory influences of the metabolites and even additional activities, as in the case of TAs.

### 6.3 Decarboxylation as an inactivation mechanism

The possibility exists that THs are also biologically decarboxylated by aromatic amino acid decarboxylase to create biogenic amine-like neuroactive compounds, such as TAMs (5, 6). To support this notion, a purified form of aromatic amino acid decarboxylase was used. However, it was not able to synthesize TAMs. This experiment further led to search for a TH-specific decarboxylase. A role of ornithine decarboxylase for this *in vitro* decarboxylation has been hypothesized (151). For example, T4, T3, T2, T1 can produce biogenic amine-like decarboxylated biologically available compounds, such as TAMs (T4AM, T3AM, three isomers of T2AM and 3-T1AM or 3’-T1AM) using specific enzymes. T3AM can further be deiodinated by specific deiodinases to produce the three isomers of T2-amine followed by the next step deiodination to 3-T1AM or 3’T1AM. Eventually, 3-T1AM can be finally completely deiodinated to form thyronamine (T0AM) which is much less potent than 3-T1AM.

### 6.4 Alternate metabolic pathways as inactivation mechanisms

As early as the 1950s, it was known that sulfated THs can be found in blood plasma (152). THs are rapidly sulfated and the resulting thyroxine sulfate (T4S) and triiodothyronine sulfate (T3S) are dramatically more sensitive to deiodination of the tyrosyl ring and less sensitive to phenolic ring deiodination, as compared to non-sulfated THs (153–155). The tyrosyl deiodination is generally associated with less potent THs. Sulfation may be a pathway to make the THs less potent and more water-soluble, thereby targeting the less-active sulfated compounds for excretion in bile or urine.

Glucuronidated THs are also found in bile, indicating further that conjugation of THs is important for degradation and excretion of the compounds (155).

After administration of 3-T1AM, the TAM is rapidly converted to N- and O-acetylated compounds (156) which are not active to regulate metabolism or body temperature (157).

Additionally, the ether linkage of the THs and TAMs is susceptible to cleavage by peroxidases, completely abolishing the activities of the compounds. However, cleavage of ether linkages is not a major pathway of TH and TAM metabolism under normal conditions (155).

## 7 Conclusions

It seems likely that some of the effects ascribed to THs might be actually due to TAMs (or TAs), subsequent to metabolism (52) (**Figure 4**). Other derivatives of TH could, in fact, act as neurotransmitters. THs, TAMs and TAs have pleiotropic actions. THs and TAMs generally have opposite effects, such that THs increase body temperature and TAMs decrease it (8). One thought is that the metabolism to TAMs could limit the timescale of the actions of THs. For example, after incubating H9c2 cells with 50 nM T3, 1.8 nM 3-T1AM accumulates intracellularly by 20 min (24). TA effects can easily be discriminated from those of the other metabolites, since they are likely related to histaminergic neurotransmission (140).

U-shaped (or inverted U-shaped, depending on the measure) activity is noted in many of the effects of TH, TAM or TA. This type of relationship is quite common in biological systems with differing mechanisms of action at low and high doses of active agent (158). One explanation for the observation is that the high doses of agent activate compensatory mechanisms (158).

With regard to the neurotransmitter-like actions of THs and metabolites, the compounds are clearly present in neural tissue, and concentrated above blood levels in synaptosomes. However, the release of the compounds has not yet been unequivocally demonstrated, possibly due to technical issues in measuring low concentrations of the hormones. This area needs further investigation. The THs and TAMs bind to high-affinity and saturable recognition sites of many different types and affinities. Here, the direct binding of THs to membrane sites is higher in affinity (nM) as compared to modulatory effects (at µM concentrations) on established neurotransmitter receptors. The latter effects resemble those of neurosteroids, and, even if not physiological in action, may be useful clinically. The THs and metabolites trigger numerous effector mechanisms, in keeping with a highly pleiotropic action of the compounds. Many of these effectors have short time courses or are present in preparations without nuclei, thereby indicating a non-canonical action. The inactivation mechanisms involve numerous metabolic pathways, some of which leave the compounds with new activities. However, the end-point of the combined pathways is excretion of the products in urine or feces, thereby completely inactivating the original compounds. In all, the definition of THs and metabolites as neurotransmitters, while attractive, is still incomplete and further research is required.

There are several areas of future research that would greatly benefit the further analysis of the hypothesis that THs or their derivatives are putative neurotransmitters. Of particular importance is the determination of the release of TH or derivatives from neural tissue or cells. In this respect, it will be of key importance to replicate the finding of Mason et al. (15) showing that Ca^2+^-dependent depolarization-induced release of TH or derivatives occurs. Mouse knock-out studies could help indicate the relevance of the various enzymes in the processes involved. Advanced microscopy techniques can give details of the localization and patterns of movement of THs and derivatives.

## Author contributions

Both JM and PS contributed to the conception of the manuscript and the drafting and revising of the text. JM and PS each approve of the publication of the manuscript and accept responsibility for the accuracy of the statements therein. All authors contributed to the article and approved the submitted version.
